# Power Distribution Internet of Things Security Risk Evaluation Based on Combined Weighting and Cloud Model

**DOI:** 10.3390/e28040433

**Published:** 2026-04-12

**Authors:** Li Peng, Jiahai Tu, Siyuan Cai, Deng Chen

**Affiliations:** 1 School of Artificial Intelligence, Hubei Open University, Wuhan 430074, China; pengli@hbou.edu.cn (L.P.); tujiahai@hbou.edu.cn (J.T.); 2 Engineering Research Center of Integration and Application of Digital Learning Technology, Ministry of Education, Beijing 100039, China; 3 Hubei Province Key Laboratory of Intelligent Robot, Wuhan Institute of Technology, Wuhan 430205, China

**Keywords:** power distribution internet of things network security, security risk evaluation, combined weighting, analytic hierarchy process, entropy weight method, cloud model, the comprehensive evaluation cloud

## Abstract

With the interconnection and intercommunication of Internet of Things (IoT) devices, the security risks of Power Distribution Internet of Things (PDIoT) systems have increased accordingly. How to monitor and assess these risks has become a key issue for advancing the construction and implementation of PDIoT. The traditional security evaluation methods mostly adopt a single weighting method and membership function, which are highly susceptible to subjective factors and have the characteristics of fuzziness and uncertainty. To address the problems, this paper proposes a security risk evaluation method of the PDIoT based on a combined weighting and cloud model. We first analyze the factors of security risks in PDIoT systems. A security evaluation index system for PDIoT was established based on factors from three aspects: the perception layer, network layer, and application layer, including 3 first-level indicators and 16 second-level indicators. Then, the analytic hierarchy process (AHP) and entropy weight method (EWM) are adopted for combined weighting to optimize the weight of each index; the cloud model is employed to calculate the standard evaluation and the comprehensive evaluation cloud. Subsequently, validity verification and cloud similarity calculation are performed to get the security state of the PDIoT. Finally the security evaluation level of the PDIoT system is obtained. An empirical test was conducted by taking the PDIoT of Meizhou Power Supply Bureau of Guangdong Power Grid as an example. The experimental results show that our method has better result distinguishability than the other three classical methods, allowing risk levels to be identified more clearly and intuitively.

## 1. Introduction

The Power Distribution Internet of Things (PDIoT) is an important component of the ubiquitous power Internet of Things, and it is a new type of distribution network state generated by the fusion of distribution technology and Internet of Things (IoT) technology [1,2]. The PDIoT has the characteristics of a complex network structure, a cloud-based master station, IoT-enabled terminal devices, and flexible services. Consequently, compared with traditional power distribution monitoring systems, the PDIoT faces greater potential security risks.

In recent years, the network security situation has become increasingly severe, and the number of security incidents of IoT and industrial control systems (ICS) has increased year by year [3]. The security risks of PDIoT can be divided into three categories. (1) Sensors: they are prone to damage due to natural or human factors, and their pluggable expansion modules can also easily trigger malicious attacks on nodes and servers. (2) Networks: with a wide exposure surface and various data types, there are many vulnerable nodes that are difficult to monitor comprehensively and timely, leading to complex security risks. (3) Terminal devices: interfaces of user-side clients generally have vulnerabilities. Moreover, due to the large number of users and imperfect identity and permission management, there is a high risk of privacy leakage.

At present, researchers mainly use three methods to assess the security of PDIoT, namely qualitative analysis, quantitative analysis, and the combination of qualitative and quantitative [4]. The combined qualitative and quantitative method, which has both subjectivity and quantitative analysis, has been widely adopted by researchers. Relevant methods include the AHP, fuzzy comprehensive evaluation method, and risk assessment model etc. For example, Wang et al. [5] proposed an evaluation and analysis method of uninterruptible operation capacity in a distribution network based on the AHP-delphi method. Sun et al. [6] proposed a reliable security risk assessment method based on AHP and an improved multi-objective particle swarm optimization (IMPSO) algorithm. Chen et al. [7] proposed a Bayesian-attack-graph-based security assessment method, the Bayesian networks are used to define and assess the potential threats that attackers may pose to different system infrastructures, thus adding the objective dimension for the evaluation. Xiao et al. [8] use principal component analysis and fuzzy comprehensive evaluation to evaluate the distribution network status in five dimensions, namely safety, economy, effectiveness, adaptability and quality. Markovic-Petrovic et al. [9] proposed a new method for security risk evaluation that considers both subjective and objective factors, which reduces the subjectivity of the evaluation. Tang [10] optimizes indicator weights by combining the analytic network process and the entropy weight method to establish an index system to evaluate the green development level of urban rail transit enterprises. Yang et al. [11] proposed an evaluation method based on EWM and the fuzzy comprehensive valuation method for the Low-Voltage distribution transformer areas. Zhou et al. [12] proposed to use the EWM and AHP model to determine the safety level of the electric distribution network. The set pair analysis (SPA) and variable fuzzy set theory (VFS) were used to reduce the analysis error caused by the fuzziness and uncertainty of the system. Cai et al. [13] proposed to use the EWM and cloud model to assess the security risk of the PDIoT; the cloud model is employed to calculate the affiliation degree and eigenvalue of each evaluation index, and the security risk status of the distribution Internet of Things is intuitively presented.

Most of the aforementioned methods adopt a single weight and membership function in the evaluation process. The membership function describes the degree to which an object belongs to a certain definition; it determines the definition scope of an object by means of fuzzy information processing formulas or corresponding weight coefficients given by experts based on practical experience, which involves strong subjective factors. Moreover, when dealing with fuzzy phenomena, the membership function converts fuzzy problems into precise problems, failing to fully reflect the uncertainty in the evaluation process and thus leading to inaccurate results. For instance, the AHP relies excessively on the evaluator’s subjectivity and is highly susceptible to subjective influences. Although the EWM reduces subjectivity in the evaluation process, it lacks the support of prior knowledge, resulting in calculation results that deviate from reality. Two methods suffer from the problem of imprecise evaluation. The method in Cai et al. [13] adopts the EWM for weight calculation to determine the security level, which has limitations in addressing the randomness issue in PDIoT security evaluation. In contrast, our method combines the AHP with EWM for combined weight assignment for security level evaluation. Specifically, AHP is used to capture experts’ subjective experience, while EWM reflects the objective information of indicator data. Compared with the single EWM adopted in Cai et al. [13], our combined weight assignment makes the weight results more reasonable.

The cloud model is a mathematical model that integrates probability theory and fuzzy mathematics to realize the mutual conversion between qualitative and quantitative data. As an evaluation method, it can effectively integrate the fuzziness and randomness of qualitative concepts and achieve the transformation between qualitative concepts and their quantitative expressions. Compared with membership functions, it has better universality and a stronger ability to describe uncertain problems [14]. Therefore, our paper proposed a security risk evaluation method for PDIoT systems based on combined weighting and a cloud model.

Our method first comprehensively considers factors from three aspects (sensors, networks, and terminal devices) to establish a PDIoT security evaluation index system, which includes three first-level indicators (risk of perception layer, risk of network layer, and risk of application layer) and 16 second-level indicators. Then, it adopts a subjective–objective combined weighting method integrating AHP and EWM to calculate the weights of evaluation indicators. Next, it generates the standard and comprehensive evaluation cloud via cloud generators. Finally, it conducts validity verification and similarity calculation in the comprehensive cloud to obtain the risk evaluation results of PDIoT. Our method overcomes the problem of information loss caused by a single weighting method, and the introduction of the cloud model enables it to address uncertain problems more comprehensively and objectively than traditional membership functions, which provides a new approach for the research on security risk evaluation of PDIoT systems.

We took the PDIoT system of Meizhou Power Supply Bureau of Guangdong Power Grid as the research object; we adopted our method to conduct a security risk evaluation on the PDIoT system. The evaluation results indicate that the security risk level of the PDIoT in this area is in a “Low Risk” state, among which the security of the perception layer and application layer is relatively good, while the security of the network layer is slightly inadequate. The overall evaluation results are consistent with the actual situation.

The main contributions of the paper are as follows:

1. We proposed a novel security evaluation method for PDIoT. It calculates the weights of evaluation indicators through integrating subjective and objective methods and employs the cloud model to realize the interconversion between qualitative and quantitative evaluation indicators, making the evaluation results more intuitive and accurate.
2. We verified the validity of the cloud model characteristic parameters of the PDIoT to avoid the cloud image atomization phenomenon during the evaluation process, which makes the evaluation results more reliable.
3. We conducted the similarity analysis on the comprehensive cloud of the PDIoT and calculated the security risk level of the PDIoT.

## 2. The Overall Framework

**Figure 1:**
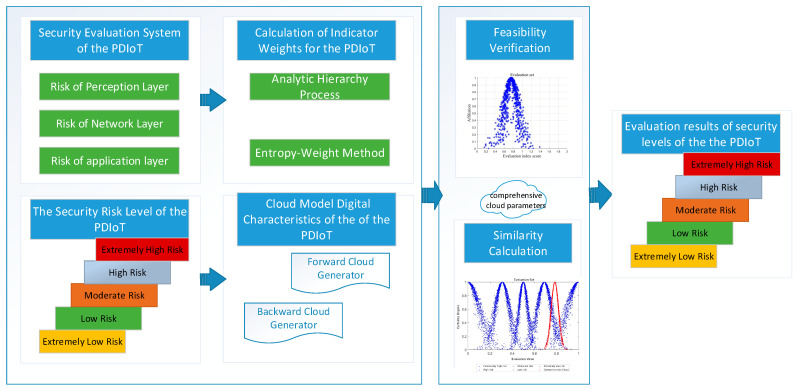
The security evaluation system of the PDIoT based on combined weighting and cloud model. Based on the three-layer architecture and the five-level security grade structure of the PDIoT, we get the indicator combined weights and the digital characteristics of the cloud model. Then, through feasibility verification and similarity calculation, the final security level evaluation results of the PDIoT are obtained.

The security evaluation system of the PDIoT was first established based on the three-layer architecture consisting of the perception layer, network layer and application layer. Then, the optimal weights of each indicator were calculated via a combined weighting method by integrating AHP and EWM. Further, the security levels of the PDIoT were classified into five grades, namely Extremely Low Risk, Low Risk, Moderate Risk, High Risk and Extremely High Risk. The standard evaluation cloud and comprehensive evaluation cloud were computed by the forward cloud generator algorithm and the backward cloud generator algorithm combined with the weights of each indicator. Finally, after conducting validity verification and similarity calculation on the comprehensive evaluation cloud, a scientific and reasonable security evaluation grade of the PDIoT can be obtained, as shown in Figure 1.

## 3. Construction of Security Evaluation Index of DPIoT

**Figure 2:**
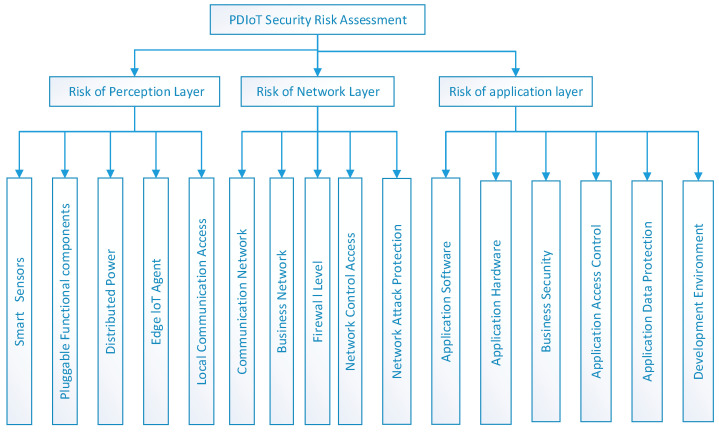
The indicators for the three-layer architecture of PDIoT. The first level consists of three first indicators, namely the perception layer risk, the risk of network layer, and the risk of application layer. The second level includes 16 sub-indicators. Specifically, the risk of the perception layer covers smart sensors, pluggable functional components, distributed power, edge IoT agent local, and communication access. The risk of the network layer covers the communication network, the business network, firewall level, network control access, and network attack protection. The risk of the application layer covers the application software, application hardware, business security management, application access control, application data protection, and development environment security.

In accordance with the principles of scientificity, comprehensiveness, systematicity, and practicality [15,16], by referring to the architectures of other IoT systems and the characteristics of the PDIoT [17], and considering its security weaknesses in three aspects—sensors, networks, and terminal devices—this paper divided the security risk of PDIoT into three layers, namely the risk of perception layer, risk of network layer, and risk of application layer, which are defined as the first-level evaluation indicators. With these three core indicators, 16 second-level evaluation indicators are divided based on the internal correlations among them. The risk of perception layer comprises smart sensors, pluggable functional components, distributed power, edge IoT agents and local communication access. The risk of the network layer includes the communication network, business network, firewall level, network control access, and network attack protection. The risk of the application layer covers application software, application hardware, business security, application access control, application data protection, and development environment, as shown in Figure 2.

## 4. The Combined Weights of Indicators

In our paper, the combined weighting method is adopted to calculate the weights of the evaluation indicators; this is a subjective–objective method that can eliminate the subjective–objective deviation and reduce the loss of information [18,19]. The subjective weights of each indicator are calculated by AHP; the objective weights of each indicator are calculated by EWM, and we performed subjective–objective combined weighting for each indicator by means of linear weighting.

### 4.1. The Subjective Weights Based on AHP

The AHP is a structured and systematic multi-criteria decision-making method proposed by Thomas L. Saaty in the 1970s. The core principle of AHP involves decomposing a complex decision problem into a hierarchical structure [9]. By introducing a 1–9 scaling method, experts conduct pairwise comparisons of elements within each level to construct judgment matrices. This process transforms subjective human judgments into quantitative numerical forms. Subsequently, the relative weights of the factors are determined by calculating the eigenvectors of the judgment matrices, and a consistency check is performed to ensure the logical coherence of the comparisons [20]. This method effectively integrates empirical judgment with mathematical models, making it particularly suitable for addressing complex issues. The calculation of subjective weights based on AHP follows the steps below.

The first step: Construct the judgment matrix. Related experts were invited to conduct pairwise comparisons among indicators at the same level. Specifically, the comparison was performed not only for the three indicators in the first level but also for the homogeneous second indicators categorized within the second level. Each expert independently provided opinions on the importance ranking of each indicator set, with the 1–9 scaling method adopted as the rating standard, as shown in Table 1. Let a certain layer of indicators in the PDIoT be denoted as $A={\left(a_{ij}\right)}_{n\times n}$, where the layer below it contains n evaluation indicators, and $a_{ij}$ represents the evaluation value obtained by pairwise comparison between indicator $a_{i}$ and indicator $a_{j}$. Then the constructed judgment matrix A is expressed as follows:

$$A=\left[\begin{array}{cc} \begin{array}{cc} a_{11} & a_{12}\\ a_{21} & a_{22} \end{array} & \begin{array}{cc} \ldots & a_{1n}\\ \ldots & a_{2n} \end{array}\\ \begin{array}{cc} \vdots & \vdots \\ a_{n1} & a_{n2} \end{array} & \begin{array}{cc} \vdots & \vdots \\ \ldots & a_{nn} \end{array} \end{array}\right]$$

The second step: Calculate the weights. The square root method is adopted to compute the weights of each judgment matrix. Let the judgment matrix be denoted as $A={\left|a_{ij}\right|}_{n\times n}$, the weight calculation formula is as follows:

(1) $$wc={\left(\prod_{j=1}^{n}a_{ij}\right)}^{1/n}/\left(\sum_{i=1}^{n}{\left(\prod_{j=1}^{n}a_{ij}\right)}^{1/n}\right),\;i=1,2,\ldots ,n$$

The third step: The consistency check. Because experts tend to have one-sided perspectives when evaluating relevant issues, it is necessary to conduct the consistency check on the judgment matrix *A*.

(2) $$\left\{\begin{array}{c} CI=\frac{\lambda_{max}-n}{n-1}\\ CR=\frac{CI}{RI} \end{array}\right.$$

where X is the judgment matrix; CI is the consistency index; CR is the consistency ratio; $\lambda_{max}$ is the maximum eigenvalue of matrix of A; n is the number of rows and columns of the judgment matrix. If CR<0.1, the judgment matrix has passed the consistency check. If CR>0.1, expert must re-score again until the consistency check is passed. The threshold of CR < 0.1 is the international universal industry standard for the consistency test of the analytic hierarchy process (AHP), which was first proposed by Saaty [21] and verified by a large number of studies in the fields of operational research and engineering evaluation.

### 4.2. The Objective Weights Based on EMP

The EWM is an objective weighting technique grounded in information theory. Information entropy is a measure used to quantify the degree of disorder or uncertainty within a system. According to the fundamental principles of information theory, a smaller information entropy for a given indicator signifies that it provides a larger amount of effective information [22]. Consequently, this indicator should be assigned a higher weight. Conversely, a larger information entropy indicates that the indicator offers less information, warranting a correspondingly lower weight. The EWM calculates weights by analyzing the inherent variability of the original data. As it relies entirely on the dispersion degree of the data itself, this method effectively mitigates the bias introduced by subjective factors [10].

The steps for calculating the indicator objective weights of the PDIoT using the EWM are presented as follows [23]:

The first step: Establish the evaluation matrix Χ. The PDIoT system involves 16 security evaluation indicators. Assuming that each indicator is assessed by n experts, ${Χ}_{ij}$ denotes the assessment value of the j-th indicator given by the i-th expert, where i=1…n, j=1…16. The assessment values of all indicators constitute the evaluation matrix X as follows.

$$X=\left[\begin{array}{cc} \begin{array}{cc} x_{11} & x_{12}\\ x_{21} & x_{22} \end{array} & \begin{array}{cc} \ldots & x_{116}\\ \ldots & x_{216} \end{array}\\ \begin{array}{cc} \vdots & \vdots \\ x_{n1} & x_{n2} \end{array} & \begin{array}{cc} \vdots & \vdots \\ \ldots & x_{n16} \end{array} \end{array}\right]$$

The second step: Standardized processing. The evaluation matrix Χ is performed standardization, and the standardization assessment matrix Y is thus obtained.

(3) $$y_{ij}=\frac{x_{ij}-\min\left(x_{j}\right)}{\max\left(x_{j}\right)-\min\left(x_{j}\right)}$$

The third step: The weight $P_{ij}$ of the jth indicator is obtained by standardization assessment matrix Y.

(4) $$P_{ij}=\frac{y_{ij}}{\sum_{i=1}^{n}y_{ij}}$$

The fourth step: The information entropy $e_{j}$ of the j-th indicator is obtained by weight $P_{ij}$.

(5) $$e_{j}=-\frac{1}{\ln n}\sum_{i=1}^{n}P_{ij}\ln\left(P_{ij}\right)$$

The fifth step: The objective weight ${ws}_{j}$ of the j-th indicator is obtained by the information entropy $e_{j}$.

(6) $${ws}_{j}=\frac{1-e_{j}}{\sum_{j-1}^{m}\left(1-e_{j}\right)}$$

### 4.3. The Calculation of Comprehensive Weights in Evaluation Indicators

The comprehensive weights of each evaluation indicator in the PDIoT system are calculated by the linear weighting method; the calculation formula is as follows:

(7) $$W=\alpha wc+\left(1-\alpha \right)ws$$

where wc, ws are the subjective weight and objective weight respectively. α is the importance degree of the subjective and objective weights. To obtain the optimal combination weight, the least squares method is used to minimize the total squared deviation between the combined weight and the subjective/objective weights. The combination coefficient α is calculated to be 0.6 [24].

## 5. The Security Risk Assessment Based on Combined Weighting and Cloud Model

We adopt the golden section method to classify the security risk levels of the PDIoT. Based on this classification, the standard evaluation cloud is established by a forward cloud generator [25]. Specifically, the forward cloud generator serves as the core algorithm that transforms qualitative security level concepts (e.g., “Extremely Low Risk”) into quantitative cloud models. By inputting the expected value, entropy, and hyper-entropy corresponding to each level, it generates a standard cloud. The standard evaluation clouds act as the benchmark cloud models for each level, which are subsequently used for similarity matching with the measured clouds. Subsequently, experts in relevant fields are invited to score the second-level indicators. Taking the scored sample data as input, a backward cloud generator is used to calculate the numerical characteristics of the cloud model. Meanwhile, combined with the indicator weights obtained by the combined weighting method, the numerical characteristics of the comprehensive cloud are computed. Finally, the validity verification of the comprehensive cloud is applied to verify the validity of the cloud to prevent the occurrence of cloud atomization, and the similarity calculation of the comprehensive cloud is utilized to determine the final security risk level of the PDIoT.

### 5.1. The Numerical Characteristics of the Cloud Model

**Figure 3:**
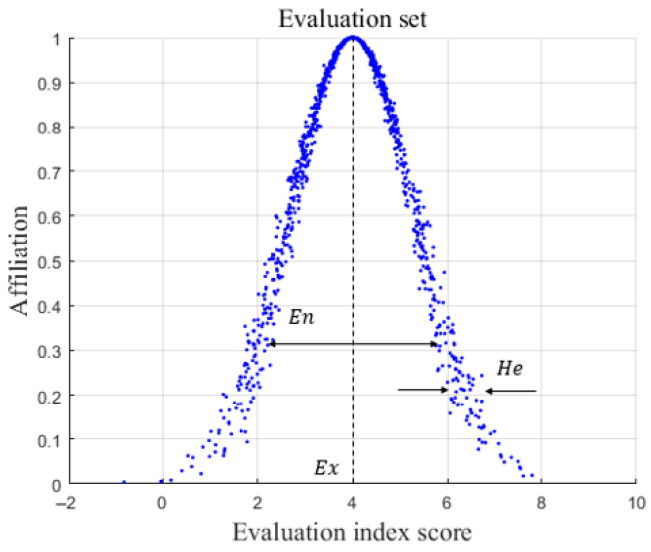
The numerical characteristics of the cloud. $E_{n}$ is the width of the cloud in the graph; $H_{e}$ corresponds to the thickness of the cloud; $E_{x}$ is the expected value.

The cloud model contains three numerical characteristics [26], namely ($E_{x},E_{n},H_{e}$), as shown in Figure 3. $E_{x}$ is the expected value; the closer the position in the cloud graph is to the expected value, the denser the cloud droplets are. $E_{x}$ is the peak of cloud in the graph; $E_{n}$ is the entropy, which measures the uncertainty of expectations, indicates the acceptable degree of fuzziness, and also reflects the dispersion degree of cloud droplets. $E_{n}$ is the width of the cloud in the graph; $H_{e}$ is the hyper-entropy, which measures the uncertainty of entropy, reveals the relationship between fuzziness and randomness, and $H_{e}$ corresponds to the thickness of the cloud.

### 5.2. Cloud Generator

The cloud generator is a tool for realizing the qualitative and quantitative transformation of the cloud model, which can be divided into the forward cloud generator and the backward cloud generator. The forward cloud generator generates the evaluation cloud droplets according to the numerical characteristics $\left(E_{x},E_{n},H_{e}\right)$. The backward cloud generator converts the risk evaluation of the PDIoT into the numerical characteristics of the cloud. The calculation steps [27] of the cloud generator are shown in Table 2.

Where $E_{x}$ is the expected value of the cloud, En is the entropy of the cloud, He is the hyper-entropy of the cloud, Enn is the cloud entropy value of the indicator after the next iteration, and $x_{i}$ is the *i*-th cloud droplet in the domain. $\mu_{A}\left(x_{i}\right)$ is the certainty degree, $\bar{X}$ is the sample mean, and $S^{2}$ is the sample variance.

### 5.3. The Standard Evaluation Cloud Model

**Figure 4:**
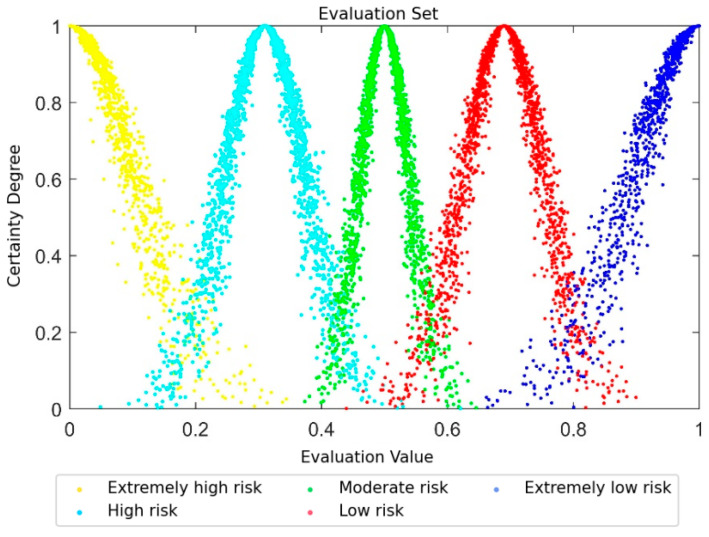
The standard evaluation cloud graph. Different color point sets represent different security levels: the yellow represents “Extremely High Risk”, the sky blue represents “High Risk”, the green represents “Moderate Risk”, the red represents “Low Risk”, and the deep blue represents “Extremely Low Risk”.

Our paper classifies the security levels of the PDIoT system and establishes a benchmark comparison chart. The PDIoT system is divided into five security levels, namely Extremely Low Risk, Low Risk, Moderate Risk, High Risk, and Extremely High Risk. The score range corresponding to each security level is defined as [0, 1], where a lower score indicates higher risk and poorer security performance. In this paper, the score set is graded using the golden section ratio algorithm, and a standard evaluation cloud is constructed. The calculation formulas are presented as follows.

(8) $${Ex}_{1}=B_{min},\;{En}_{1}=\frac{{En}_{2}}{0.618},\;{He}_{1}=\frac{{He}_{2}}{0.618}$$

(9) $${Ex}_{2}={Ex}_{3}-\frac{0.382\times (B_{max}-B_{min})}{2},\;{En}_{2}=\frac{0.382\times (B_{max}-B_{min})}{2},\;{He}_{2}=\frac{{He}_{3}}{0.618}$$

(10) $${Ex}_{3}=\frac{B_{max}+B_{min}}{2},\;{En}_{3}=0.618\times {En}_{4},\;{He}_{3}=0.007$$

(11) $${Ex}_{4}={Ex}_{3}+\frac{0.382\times (B_{max}-B_{min})}{2},\;{En}_{4}=\frac{0.382\times (B_{max}-B_{min})}{2},\;{He}_{4}=\frac{{He}_{3}}{0.618}$$

(12) $${Ex}_{5}=B_{max},\;{En}_{5}=\frac{{En}_{4}}{0.618},\;{He}_{5}=\frac{{He}_{4}}{0.618}$$

where $B_{min}$ and $B_{max}$ are the minimum and maximum values of the corresponding score range, i.e., 0 and 1, respectively. $\left({Ex}_{i},{En}_{i},{He}_{i}\right)\;(i=1,\;2,\;3,\;4,\;5)$ are the numerical characteristics of the cloud model corresponding to the five security levels. The ratio of the cloud numerical characteristics between adjacent evaluation levels is the golden ratio. 0.618, 0.618 and its complement 0.382 are classic and commonly used settings for grade division and standard cloud construction in the cloud model, which have been widely adopted in the foundational research of cloud model by Li et al. [28,29]; the closer they are to the center of the domain, the lower the values of entropy and hyper-entropy. Among these, ${He}_{3}$ is determined based on a specific scenario and ${He}_{3}$ is 0.007. In the cloud model, the hyper-entropy He represents the entropy of entropy and directly affects the cohesion of cloud droplets. According to the research of Xia et al. [30], the condition *He* < *En*/3 must be satisfied; otherwise, the cloud droplets will be excessively dispersed, leading to a “fuzzification” phenomenon. In this paper, ${He}_{3}$ is set to 0.007, as shown in Figure 4. This value not only ensures the stability of the middle-level cloud but also provides a reasonable benchmark for the *He* values of other levels.

The numerical characteristics of the standard evaluation cloud are shown in Table 3. The table lists the cloud numerical characteristics (Ex,En,He) corresponding to the five security risk levels. Taking the “High Risk” level as an example, the expected value Ex=0.31, entropy value En=0.064, and hyper-entropy value He=0.018.

The standard evaluation cloud can be plotted based on the calculation results in Table 3, as shown in Figure 4. The normal cloud in different colors represents different security risk levels, which are Extremely Low Risk, Low Risk, Moderate Risk, High Risk, and Extremely High Risk from right to left.

### 5.4. The Comprehensive Evaluation Cloud Model

First, the numerical characteristics of the cloud model for second-level evaluation indicators are calculated by the backward cloud generator. Then, the corresponding numerical characteristics of first-level indicators and the comprehensive cloud model are derived according to Formulas (13)–(15). These characteristics are input into the forward cloud generator to obtain the comprehensive security risk assessment cloud of the PDIoT system. By comparing this graph with the standard evaluation cloud, a preliminary judgment can be made on the current security risk level of the PDIoT system.

(13) $$Ex=\frac{\sum_{i=1}^{n}w_{i}\times {Ex}_{i}}{\sum_{i=1}^{n}w_{i}}$$

(14) $$En=\frac{\sum_{i=1}^{n}w_{i}^{2}\times {En}_{i}}{\sum_{i=1}^{n}w_{i}^{2}}$$

(15) $$He=\frac{\sum_{i=1}^{n}w_{i}^{2}\times {He}_{i}}{\sum_{i=1}^{n}w_{i}^{2}}$$

where $w_{i}$ is the combined weights of each indicator; $\left({Ex}_{i},{En}_{i},{He}_{i}\right)$ is the cloud numerical characteristics of each indicator; and n is the number of indicators at the corresponding level. $\left(E_{x},E_{n},H_{e}\right)$ refers to the cloud numerical characteristics of the corresponding first-level indicator and the comprehensive evaluation cloud.

### 5.5. Validity Verification of Comprehensive Evaluation Cloud

**Figure 5:**
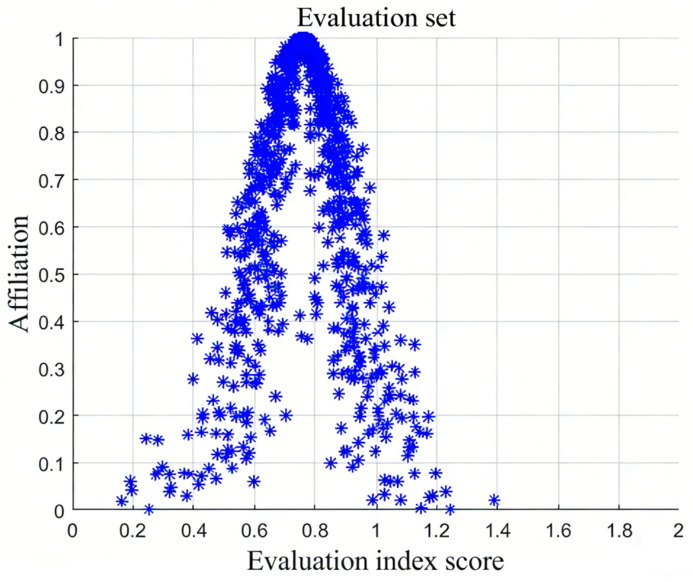
Cloud model in an atomized state.

According to the atomization characteristics of the cloud model and the 3δ principle of normal functions [30], when He<En/3, the cloud model is in a sound state, where 99.8% of the cloud droplets fall within the range between the minimum boundary curve $y=exp[-\frac{{\left(x-Ex\right)}^{2}}{2{\left(En-3He\right)}^{2}}]$ and the maximum boundary curve $y=exp[-\frac{{\left(x-Ex\right)}^{2}}{2{\left(En+3He\right)}^{2}}]$. When He>En/3, the cloud model suffers from severe atomization and it needs to be reconstructed, as shown in Figure 5. The cloud droplets of this normal cloud show a sparse distribution. Therefore, En/3 is defined as the atomization point of the standard evaluation cloud model, and the cloud models that fail to meet the validity criteria shall be re-evaluated by the experts.

### 5.6. Similarity Calculation of the Comprehensive Evaluation Cloud

After generating the standard cloud and comprehensive cloud, it may be impossible to determine the risk levels due to the similarity of the evaluation results on the cloud figures. Therefore, we adopt the IkLCM algorithm [14] to calculate the cloud similarity. This algorithm combines the modified expected curve with the Kullback–Leibler (KL) divergence, and comprehensively considers the effects of expectation, entropy, and hyper-entropy in determining the risk levels of the PDIoT. Assuming that the numerical characteristics of the standard evaluation cloud $S_{i}$ are $\left({Ex}_{i},{En}_{i},{He}_{i}\right)$ and those of the comprehensive cloud $S_{j}$ are $\left({Ex}_{j},{En}_{j},{He}_{j}\right)$, the divergence calculation formula between the two normal clouds is as follows:

(16) $$D\left(S_{i},S_{j}\right)=\frac{1}{2}\times \left[{\left({Ex}_{i}-{Ex}_{j}\right)}^{2}+\left(\sigma_{i}^{2}+\sigma_{j}^{2}\right)\right]\times \left(\frac{1}{\sigma_{i}^{2}+\sigma_{j}^{2})}\right)-2$$

where $\sigma_{i}^{2}={En}_{i}^{2}+{He}_{i}^{2},\sigma_{j}^{2}={En}_{j}^{2}+{He}_{j}^{2}$. In comparison with similarity functions, an exponential function is introduced to constrain the similarity value within the range of [0, 1]; the exponential function $sim(S_{i},S_{j})$ is as follows:

$$sim\left(S_{i},S_{j}\right)=exp(-D(S_{i},S_{j}))$$

## 6. Experiment

We present the experimental verification and comparative analysis. First, the specific evaluation process is elaborated, including the selection of research objects and the implementation of index scoring and importance judgment based on the established evaluation index system. Subsequently, the security evaluation results of the system are analyzed and discussed with the support of cloud model visualization. Finally, to further demonstrate the superiority of our method, comparative experiments are conducted with three classical evaluation methods, and the results are systematically compared and analyzed to verify the stability and rationality of the evaluation outcomes.

### 6.1. Security Evaluation Process of the PDIoT

In this paper, the PDIoT system of Meizhou Power Supply Bureau of Guangdong Power Grid is adopted as the research object to verify its feasibility. Based on the security evaluation index system constructed in Figure 1, relevant experts were invited to form the evaluation panel, who scored each index and judged its importance respectively. Senior engineers from the power supply bureaus were invited to participate in the survey, including 2 Deputy Chief Engineers and 2 Technical Department Engineers from this bureau, as well as 1 Deputy Chief Engineer and 2 Technical Department Engineers from each of the other two bureaus, totaling 10 participants. Combining practical conditions and referring to corresponding scoring rules, the participants judged the importance and scored each indicator. Both Deputy Chief Engineers and grassroots department engineers possess distinct advantages. After conducting consistency tests on the judgment matrices of the 10 participants, geometric mean calculations were performed on the matrices, yielding the final judgment matrices. The average judgment matrices for each primary indicator A and the secondary indicators $B_{1}$, $B_{2}$, $B_{3}$, under each primary indicator are as follows:

$$A=\;\left[\begin{array}{ccc} 1 & 1.3 & 0.95\\ 1/1.3 & 1 & 0.75\\ 1/0.95 & 1/0.75 & 1 \end{array}\right]$$

$$B_{1}=\left[\begin{array}{ccccc} 1 & 1.15 & 1.91 & 0.63 & 2.48\\ 1/1.15 & 1 & 1.66 & 0.55 & 2.16\\ 1/1.91 & 1/1.66 & 1 & 0.33 & 1.30\\ 1/0.63 & 1/0.55 & 1/0.33 & 1 & 3.94\\ 1/2.48 & 1/2.16 & 1/1.30 & 1/3.94 & 1 \end{array}\right]\;B_{2}=\left[\begin{array}{ccccc} 1 & 5.88 & 1.05 & 0.59 & 0.58\\ 1/5.88 & 1 & 0.18 & 0.1 & 0.10\\ 1/1.05 & 1/0.18 & 1 & 0.56 & 0.55\\ 1/0.59 & 1/0.10 & 1/0.56 & 1 & 0.98\\ 1/0.58 & 1/0.10 & 1/0.55 & 1/0.98 & 1 \end{array}\right]\;B_{3}=\left[\begin{array}{cccccc} 1 & 0.82 & 1.33 & 7.30 & 1.66 & 0.79\\ 1/0.82 & 1 & 1.62 & 8.90 & 2.02 & 0.96\\ 1/1.33 & 1/1.62 & 1 & 5.49 & 1.25 & 0.59\\ 1/7.30 & 1/8.90 & 1/5.49 & 1 & 0.23 & 0.11\\ 1/1.66 & 1/2.02 & 1/1.25 & 1/0.23 & 1 & 0.48\\ 1/0.79 & 1/0.96 & 1/0.59 & 1/0.11 & 1/0.48 & 1 \end{array}\right]$$

The scores from the 10 experts were collected, and the results are shown in Table 4.

In accordance with the AHP, judgment matrices of the first-level indices and second-level indices were constructed separately. The eigenvalues and eigenvectors of each judgment matrix were calculated. After passing the consistency test, the subjective weights of the evaluation indices at all levels of the PDIoT system were obtained by normalizing the eigenvectors. Based on the scoring results of the relevant experts, the objective weights of each evaluation index in the three layers were derived using the EWM, the objective weights of all second-level indices were summed up to obtain the objective weights of the first-level indices. Combining the results obtained above, the combined weights of the indices at all levels were calculated according to Equation (7), with the results shown in Table 5 and Table 6. Table 5 lists the subjective weights, objective weights and combined weights of all second-level indices, while Table 6 presents the subjective weights, objective weights and combined weights of all first-level indices, namely, the risks of the perception layer, network layer and application layer.

The scoring results of the 16 indices by the expert panel were converted into quantitative data as the input of the backward cloud generator, and the numerical characteristics of the cloud model for each index were calculated. Then, the combined weights of the second-level indices were integrated with the numerical characteristic values of the cloud, and the numerical characteristics of the cloud model for the first-level indices were calculated by Equations (12)–(14), as shown in Table 7. Further, the numerical characteristics of the cloud model integrated with the index weights of the first-level indices; the numerical characteristics of the comprehensive cloud model were calculated as (0.758, 0.033, 0.0018).

**Figure 6:**
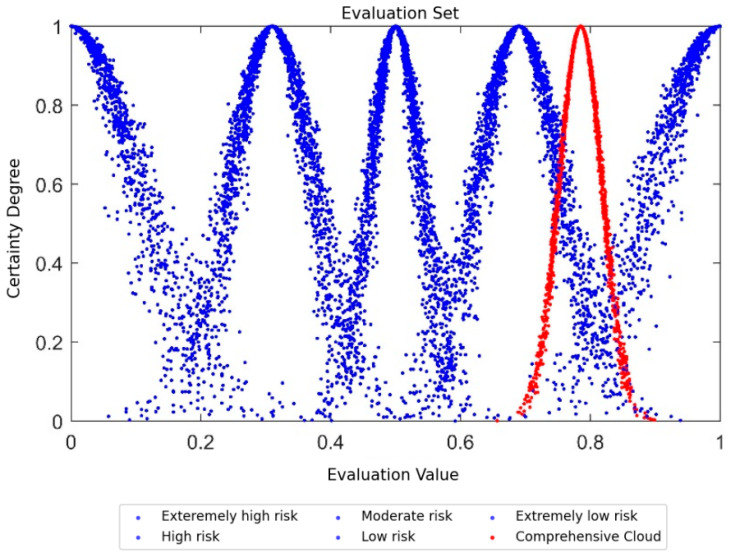
Comprehensive evaluation cloud of PDIoT. The figure compares the comprehensive evaluation cloud diagram with the standard evaluation cloud diagram to judge the security level of the comprehensive cloud. The red represents the comprehensive evaluation cloud, and the blue represents the standard evaluation cloud.

Validity verification was performed on the computing results of indices at all levels. It was found that the numerical characteristics of the cloud model for all indices satisfy He<En/3, which means that the evaluation results are valid and reliable. Based on the numerical characteristics of the comprehensive cloud model for PDIoT, the corresponding comprehensive evaluation cloud was generated and compared with the standard evaluation cloud in Figure 4; the comparison results are shown in Figure 6. The blue normal cloud is the standard evaluation cloud, while the red normal cloud is the comprehensive evaluation cloud of the PDIoT. It can be observed that the comprehensive evaluation cloud of the PDIoT lies between the “Extremely Low Risk” and “Low Risk” levels. Furthermore, similarity analysis on the comprehensive cloud model reveals that the comprehensive evaluation cloud of the PDIoT has the highest similarity to the Low Risk cloud. Thus, it can be concluded that the comprehensive assessment grade of the PDIoT system is “Low Risk”. The power supply station was constructed in 2021. From its completion to 2024, continuous monitoring indicates that no major safety incidents have occurred at the station. Our evaluation results are consistent with the actual situation, showing good stability and rationality, which verifies the effectiveness and feasibility of our method.

### 6.2. Analysis of Evaluation Results

As can be seen from the cloud model of the PDIoT in Figure 6, the security evaluation grade of the PDIoT system in this region is at the “Low Risk” level. Moreover, based on the numerical characteristics of the cloud model for indices at all levels, it is found that the scores of the perception layer *A*_1_ and the application layer *A*_3_ are generally high. This indicates that most terminal devices of the PDIoT, such as DTUs, FTUs and smart sensors, are well maintained; the professional competence of the staff is strong; the software and hardware environment is stable; and the possibility of large-scale security incidents is low. In contrast, the scores of all indices under the network layer *A*_2_ are generally low, which reveals that the PDIoT system is relatively weak in network security management. This result is consistent with the internal security inspection findings of the system. The internal security inspection shows that due to the large scale of the PDIoT system, the vagueness and complexity of communication protocols, and the existence of numerous open network ports, information is vulnerable to interception and loss during transmission. In addition, the network firewall is weak, making the system susceptible to cyberattacks.

In response to the above problems, the following recommendations are put forward for the PDIoT system in this region: strengthen identity authentication and access control to prevent attackers from impersonating legitimate users to obtain resource access permissions [27,30,31]. Enhance firewall construction, detect and eliminate viruses in a timely manner, and ward off malicious attacks. Improve network communication protocols to ensure the confidentiality and integrity of data transmission. Continuously revise and improve various security management systems, ensure their implementation and effective execution, strengthen supervision over frontline maintenance personnel, and maintain real-time oversight of maintenance site conditions.

### 6.3. Comparative Analysis

**Figure 7:**
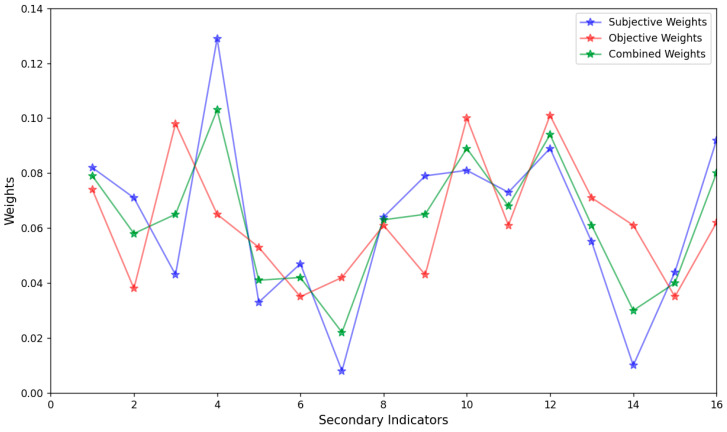
Comparison of weights of different security evaluation methods. The blue represents the subjective weights obtained by AHP, the red represents the objective weights obtained by EWM, and the green represents the combined weights generated by our algorithm. The horizontal axis represents the serial numbers of indicators, and the vertical axis represents the weights of the corresponding indicators.

Three evaluation methods, namely the gray fuzzy evaluation method [32], AHP-fuzzy comprehensive evaluation method [33], and entropy weight-cloud model method [13], were selected for comparison with our method. The comparison results are presented in Table 8 and Figure 7.

As can be seen from Table 8, the proposed method exhibits superior result distinguishability compared with the other three classical methods. Specifically, the membership degree distribution of the proposed method presents absolute concentration; it only shows a non-zero membership degree of 0.30 at the “Low Risk” level, while the membership degrees at the four levels of “Extremely High Risk”, “High Risk”, “Moderate Risk”, and “Extremely Low Risk” are all 0. This indicates that the method can clearly eliminate the interference of non-target risk levels and accurately lock in the actual safety status of the system. In contrast to the distribution of other methods, the Gray fuzzy evaluation method has non-zero membership degrees (0.10–0.35) across all five risk levels; the AHP-fuzzy comprehensive evaluation method also covers all levels (0.14–0.33). Although the entropy weight-cloud model method has a membership degree of 0 at the High, Moderate, and Extremely High Risk levels, it still has an interference of 0.08 membership degree at the Extremely Low Risk level. The scattered membership degree characteristics of these three methods lead to ambiguity in risk level determination. However, our method significantly improves the distinguishability and accuracy of evaluation results through its completely concentrated membership degree distribution. And among the four evaluation methods, the proposed method and the entropy weight-cloud model method have visualization capabilities, while the Gray fuzzy evaluation method and the AHP-fuzzy comprehensive evaluation method do not possess visualization functions. This difference directly affects the intuitive perception effect of risk status.

Moreover, according to the comparison of weights calculation results of different security evaluation methods presented in Figure 7, the weights derived from the combined weighting method effectively reduce subjective randomness and make up for the deficiencies of the EWM. For example, the objective weight of Indicator *A*_14_ (edge IoT agent local) calculated by the EWM is 0.065 (lower information entropy caused by small data fluctuations). However, considering its business importance (the subjective weight via AHP is 0.129, which is higher than that of Indicator *A*_13_), the corrected combined weight is adjusted to 0.103. This result neither ignores the value of data nor excessively amplifies the weight of non-core indicators. For Indicator *A*_13_ (distributed power), its EWM objective weight is 0.098 (higher information entropy due to large data fluctuations), but combined with its subjective weight of 0.043, the corrected combined weight is revised to 0.065. Through the correction of combined weights, the weight of the edge IoT agent local indicator is effectively increased and the weight of the distributed power indicator is reduced, resulting in the weight of the edge IoT agent local being greater than that of the distributed power. This indicates that the combined weighting method effectively compensates for the limitation of EWM. These findings indicate that our method is reasonable and effective. Compared with other methods, our paper has higher discriminability. It not only mitigates the drawbacks of single weighting methods but also addresses the problems of randomness and fuzziness in the assessment process, as well as the issues regarding the effectiveness of the cloud model and the refinement of membership functions.

In summary, our method exhibits superior result distinguishability compared with the other three classical methods, enabling clearer and more intuitive identification of risk levels. In addition, our method not only alleviates the inherent drawbacks of single weighting methods but also effectively addresses the issues of randomness and fuzziness in the security assessment process. Therefore, it provides a more reasonable, reliable and practical solution for the security risk assessment of Power Distribution Internet of Things systems.

## 7. Conclusions

We propose a security evaluation method for the PDIoT based on combined weighting and a cloud model. According to the structural characteristics of the PDIoT, we construct a security evaluation system based on the perception layer, network layer, and application layer. The optimal weights of each indicator are determined by the combined weighting method that integrates the AHP and EWM, which addresses the problem of information loss caused by a single weighting function. By introducing a cloud model, a security risk evaluation model for the PDIoT is established, overcoming the fuzziness and uncertainty inherent in traditional assessment methods. Furthermore, validity verification and similarity analysis are conducted on the comprehensive cloud model, making the evaluation results more reliable and accurate. A case study is carried out on the PDIoT system of Meizhou Power Supply Bureau of Guangdong Power Grid. The characteristic parameters of the comprehensive cloud model for the PDIoT are as follows: Ex=0.758, En=0.033, He=0.0018. The results prove the validity of the cloud model. Through cloud similarity calculation, the risk assessment level of the PDIoT system is determined as “Low Risk”. This result is consistent with the actual situation of the region, verifying the effectiveness and feasibility of our method. However, if the sample size is small, the backward cloud model algorithm has a large error in hyper-entropy estimation [34]. Therefore, future research will focus on improving the backward cloud algorithm [34,35,36,37].

## Figures and Tables

**Table 1:** 1–9 scaling method.

$\mathrm{Scale}\;a_{ij}$	Mean
1	Indicator i is of equal importance to Indicator j.
2	Indicator i is slightly more important than Indicator j.
9	Indicator i is significantly more important than Indicator j.
2~8	Intermediate values between the adjacent two scales
reciprocal	Reciprocal values of the above scales

**Table 2:** The calculation steps of cloud generator.

The Forward Cloud Generator	The Backward Cloud Generator
① $Enn\sim N(En,{He}^{2})$	$\bar{X}=\frac{1}{n}\sum_{i=1}^{n}x_{i}$, $S^{2}=\frac{1}{n-1}\sum_{i=1}^{n}\left(x-\bar{X}\right)$
② $x_{i}\sim N(Ex,{He}^{2})$	$Ex=\bar{X}$
③ $\mu_{A}\left(x_{i}\right)=exp(-{\left(x_{i}-Ex\right)}^{2}/\left(2{\left(Enn\right)}^{2}\right))$	$En=\sqrt{\frac{\pi }{2}}\frac{1}{n}\sum_{i=1}^{n}\left|x_{i}-\bar{X}\right|$
④ $\left(x_{i},\mu_{A}\left(x_{i}\right)\right),i=1,2,\ldots ,n$	$He=\sqrt{S^{2}-{En}^{2}}$

**Table 3:** The cloud numerical characteristics in each level.

Risk Level	Cloud Numerical Characteristics
Extremely High Risk	(0, 0.104, 0.018)
High Risk	(0.31, 0.064, 0.011)
Moderate Risk	(0.5, 0.04, 0.007)
Low Risk	(0.69, 0.064, 0.011)
Extremely Low Risk	(1, 0.104, 0.018)

**Table 4:** The scores from the 10 experts.

	*A*_11_	*A*_12_	*A*_13_	*A*_14_	*A*_15_	*A*_21_	*A*_22_	*A*_23_	*A*_24_	*A*_25_	*A*_31_	*A*_32_	*A*_33_	*A*_34_	*A*_35_	*A*_36_
1	8.9	8.6	7.4	5.9	7.7	6.8	5.6	6.5	8.0	5.3	9.2	9.8	8.3	7.1	9.5	6.2
2	8.8	8.5	7.3	5.8	7.6	6.7	5.5	6.4	7.9	5.2	9.1	9.7	8.2	7.0	9.4	6.1
3	9.0	8.7	7.5	6.0	7.8	6.9	5.7	6.6	8.1	5.4	9.3	9.9	8.4	7.2	9.6	6.3
4	8.7	8.4	7.2	5.7	7.5	6.6	5.4	6.3	7.8	5.1	9.0	9.6	8.1	6.9	9.3	6.0
5	9.1	8.8	7.6	6.1	7.9	7.0	5.8	6.7	8.2	5.5	9.4	9.9	8.5	7.3	9.7	6.4
6	8.6	8.3	7.1	5.6	7.4	6.5	5.3	6.2	7.7	5.0	8.9	9.5	8.0	6.8	9.2	5.9
7	8.9	8.6	7.4	5.9	7.7	6.8	5.6	6.5	8.0	5.3	9.2	9.8	8.3	7.1	9.5	6.2
8	8.8	8.5	7.3	5.8	7.6	6.7	5.5	6.4	7.9	5.2	9.1	9.7	8.2	7.0	9.4	6.1
9	9.0	8.7	7.5	6.0	7.8	6.9	5.7	6.6	8.1	5.4	9.3	9.9	8.4	7.2	9.6	6.3
10	8.7	8.4	7.2	5.7	7.5	6.6	5.4	6.3	7.8	5.1	9.0	9.6	8.1	6.9	9.3	6.0

**Table 5:** Weights of each second-level index.

Index	Subjective Weights	Objective Weight	Combined Weights
*A*_11_	0.082	0.074	0.079
*A*_12_	0.071	0.038	0.058
*A*_13_	0.043	0.098	0.065
*A*_14_	0.129	0.065	0.103
*A*_15_	0.033	0.053	0.041
*A*_21_	0.047	0.035	0.042
*A*_22_	0.008	0.042	0.022
*A*_23_	0.064	0.061	0.063
*A*_24_	0.079	0.043	0.065
*A*_25_	0.081	0.100	0.089
*A*_31_	0.073	0.061	0.068
*A*_32_	0.089	0.101	0.094
*A*_33_	0.055	0.071	0.061
*A*_34_	0.010	0.061	0.030
*A*_35_	0.044	0.035	0.040
*A*_36_	0.092	0.062	0.080

**Table 6:** Weights of each first-level index.

Index	Subjective Weights	Objective Weights	Combined Weights
*A*_1_	0.358	0.328	0.346
*A*_2_	0.279	0.281	0.280
*A*_3_	0.363	0.391	0.374

**Table 7:** Cloud model numerical characteristics of first-level and second-level indices.

First-Level	Cloud Model Numerical Characteristics	Second-Level	Cloud Model Numerical Characteristics
*A*_1_	(0.759, 0.027, 0.0016)	*A*_11_	(0.833, 0.029, 0.0016)
		*A*_12_	(0.846, 0.030, 0.0017)
		*A*_13_	(0.747, 0.027, 0.0016)
		*A*_14_	(0.659, 0.031, 0.0017)
		*A*_15_	(0.760, 0.029, 0.0017)
*A*_2_	(0.639, 0.053, 0.0022)	*A*_21_	(0.697, 0.088, 0.0029)
		*A*_22_	(0.639, 0.053, 0.0022)
		*A*_23_	(0.727, 0.030, 0.0017)
		*A*_24_	(0.773, 0.038, 0.0019)
		*A*_25_	(0.557, 0.037, 0.0019)
*A*_3_	(0.834, 0.039, 0.0019)	*A*_31_	(0.860, 0.024, 0.0015)
		*A*_32_	(0.889, 0.019, 0.0014)
		*A*_33_	(0.834, 0.039, 0.0019)
		*A*_34_	(0.740, 0.023, 0.0015)
		*A*_35_	(0.833, 0.101, 0.0030)
		*A*_36_	(0.731, 0.031, 0.0017)

**Table 8:** Results of different security risk evaluation methods.

Assessment Methods	Affiliation Degree Corresponding to Different Risk Levels					Assessment Level
	Extremely High Risk	High Risk	Moderate Risk	Low Risk	Extremely Low Risk	
Gray fuzzy evaluation	0.10	0.18	0.28	0.35	0.21	Low Risk
AHP-fuzzy comprehensive evaluation	0.14	0.22	0.24	0.32	0.33	Low Risk
Entropy weight-cloud model	0	0	0	0.45	0.08	Low Risk
Our method	0	0	0	0.30	0	Low Risk

## Data Availability

The data presented in this study are available on request from the corresponding author.

## Abbreviations

The following abbreviations are used in this manuscript:

IoT	Internet of Things
PDIoT	Power Distribution Internet of Things
AHP	Analytic Hierarchy Process
EWM	Entropy Weight Method
ICS	Industrial Control System
